# An Atypical Presentation of a Saddle Embolism in the Setting of Malignancy

**DOI:** 10.7759/cureus.57215

**Published:** 2024-03-29

**Authors:** Carly Esposito, Lucas C Zadan, Admir Seferovic, Yuliya Markova-Acevedo, Enrique Urrutia

**Affiliations:** 1 Family Medicine, HCA Florida St. Petersburg Hospital, St. Petersburg, USA; 2 Graduate Medical Education, HCA Florida St. Petersburg Hospital, St. Petersburg, USA

**Keywords:** v/q scan, renal cell carcinoma (rcc), computed tomography pulmonary angiography, saddle pe, pulmonary thrombectomy

## Abstract

A 52-year-old male presented to the emergency room with acute abdominal pain. Imaging determined acute appendicitis, with an incidental finding of a renal mass. The biopsy was positive for renal cell carcinoma, and the patient underwent simultaneous appendectomy and nephrectomy. Postoperatively, the patient developed hypoxia at night with exertion, requiring oxygen supplementation. The remainder of his vital signs were stable. Due to renal function, a ventilation/perfusion (V/Q) scan was conducted, which showed a high probability of pulmonary embolism (PE). Follow-up computed tomography angiography of the chest showed a massive saddle embolism. Interventional radiology performed an uncomplicated thrombectomy, oxygen saturations improved, and the patient was discharged on apixaban.

## Introduction

This was a non-classic case of a saddle pulmonary embolism; it presented as a silent PE that was found and treated in time before hemodynamic status was compromised. While the risk factors of PE formation were present (i.e. malignancy, surgery, acute medical illness), the presentation was unusual in a textbook sense. The clinical presentation of a pulmonary embolism typically includes signs and symptoms such as dyspnea, pleuritic chest pain, cough, tachypnea, rales, and tachycardia [1]. The patient elicited none of these symptoms listed above. Additionally, the patient had a venous Doppler ultrasound of both legs that showed no evidence of thrombi. The work-up was further complicated due to a recent nephrectomy and elevated creatinine. The gold standard for diagnosing PE is a computed tomography angiogram (CTA) of the chest using intravenous contrast. However, due to the patient’s renal compromise, a multidisciplinary discussion was had to weigh the risks and benefits of using contrast. Notably, we discussed relative risk versus contraindications of using contrast in this particular case in fear of contrast-associated nephrotoxicity.

## Case presentation

**Figure 1:**
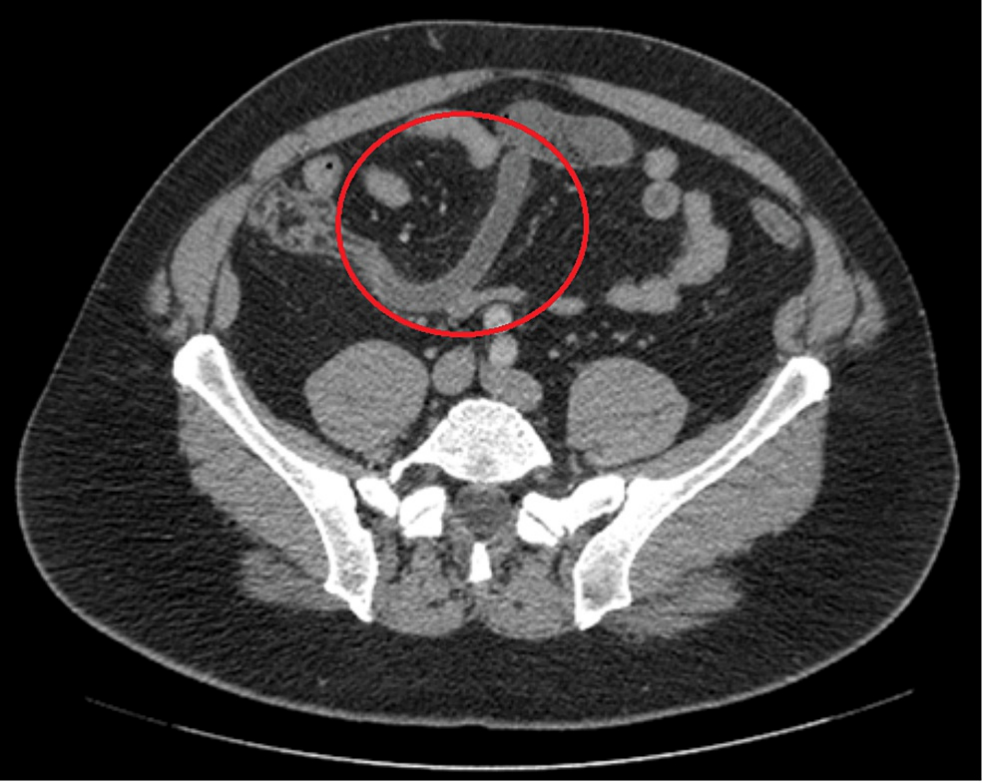
Appendicitis captured on computed tomography transverse view prior to appendectomy procedure

**Figure 2:**
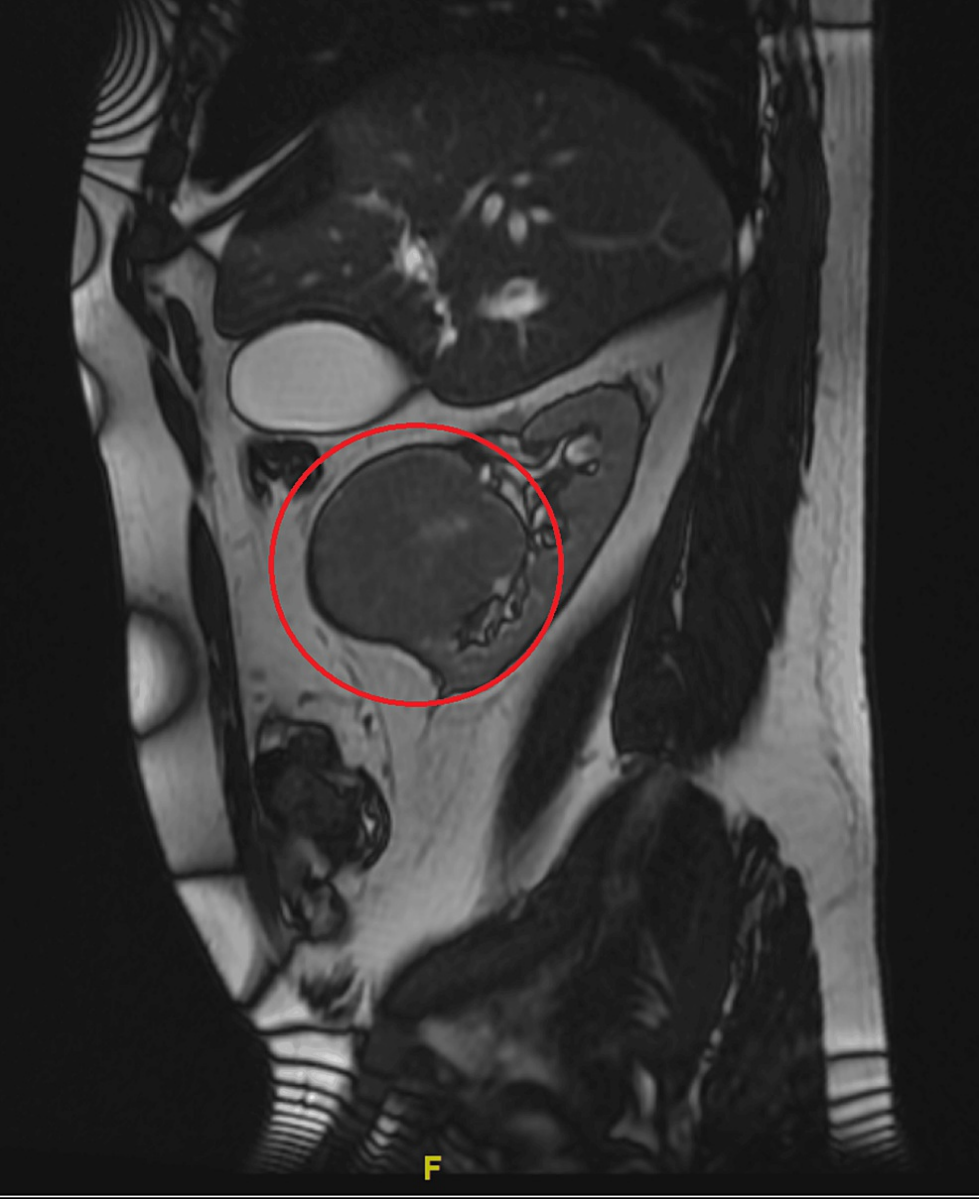
Renal cell carcinoma of the right kidney captured on magnetic resonance imaging sagittal view prior to nephrectomy procedure (red circle).

**Figure 3:**
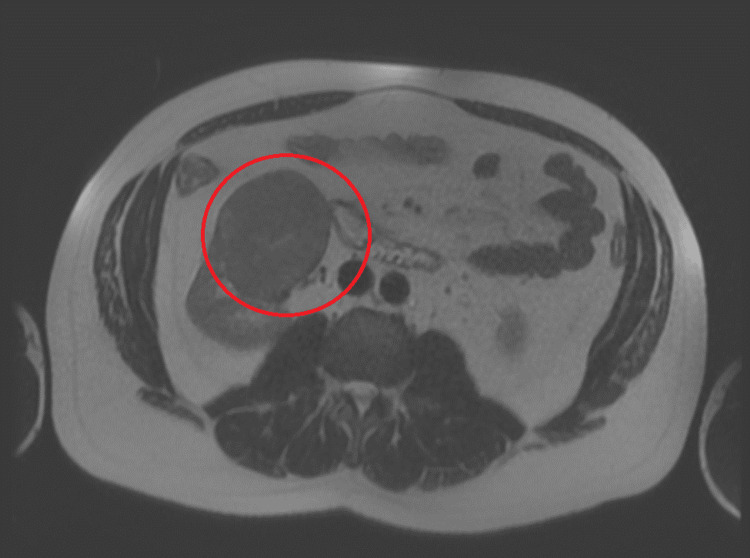
Renal cell carcinoma of the right kidney captured on magnetic resonance imaging transverse view prior to nephrectomy procedure (red circle)

**Figure 4:**
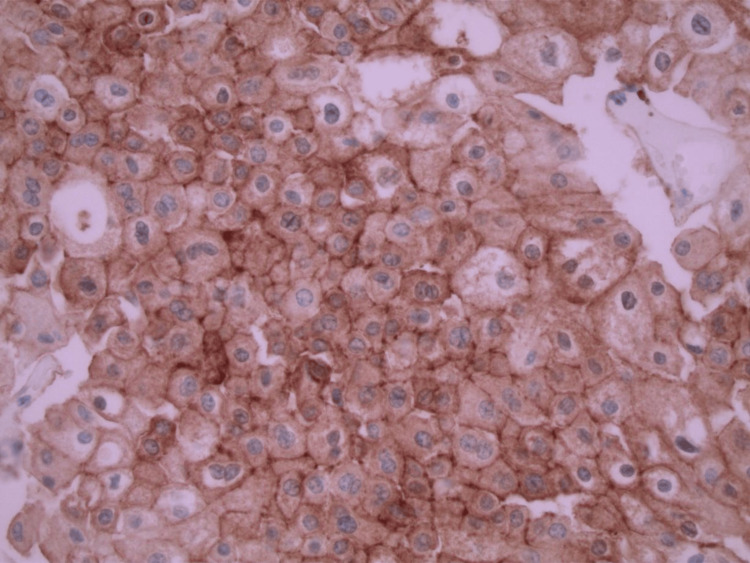
CD117 immunohistochemical marker positive for chromophobe renal cell carcinoma

**Figure 5:**
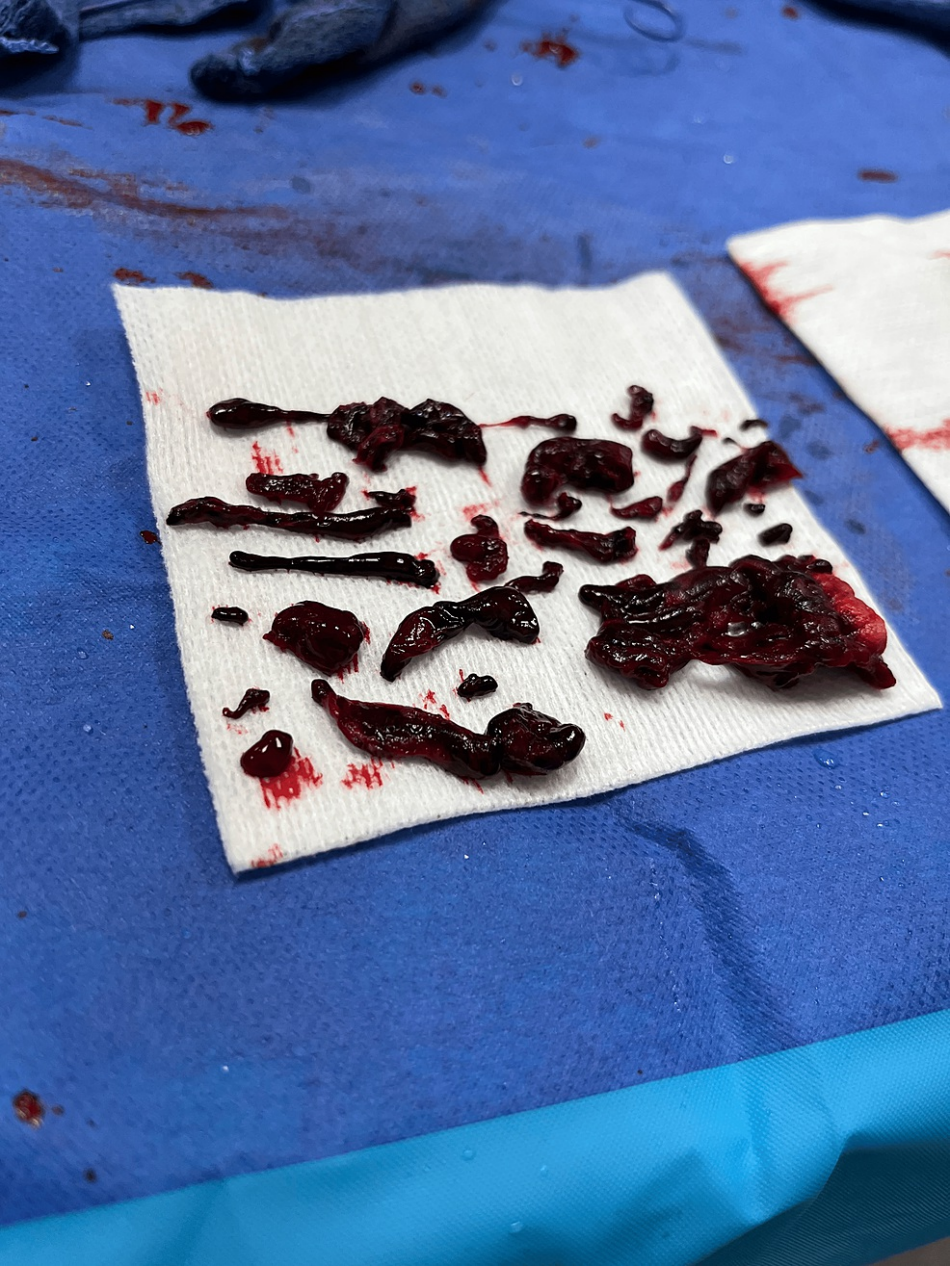
Total collected thrombi after thrombectomy procedure. These collected thrombi were all components of the patient's massive saddle embolism

**Figure 6:**
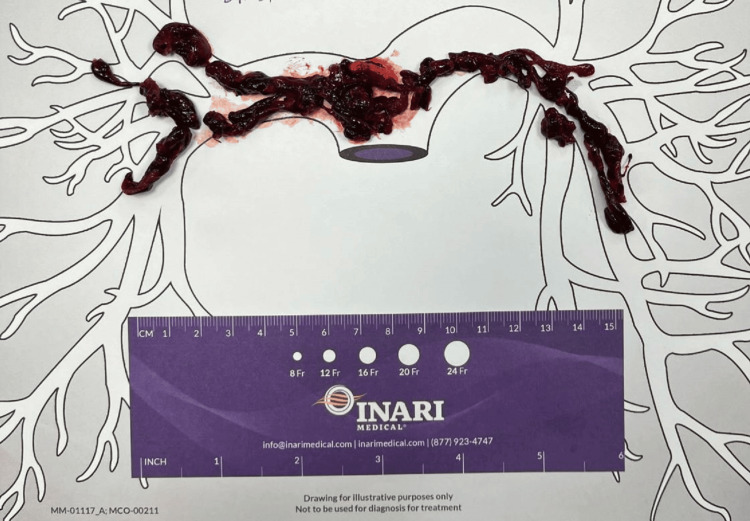
Likely location and size of pulmonary embolus after removal via thrombectomy

A 52-year-old male presented to the emergency department for evaluation of right lower quadrant abdominal pain. The patient was afebrile, hypertensive, and exhibited tenderness in the right lower quadrant upon palpation. Computed tomography (CT) of the abdomen and pelvis without contrast revealed a distended appendix with mild inflammation consistent with acute appendicitis (Figure 1). An incidental finding of a right lower renal pole solid mass measuring 8.0 x 6.9 x 7.1 cm, without cystic components or calcifications (Figure 2). An MRI of the abdomen revealed an 8.4-cm renal mass with a capsule that was hypo-enhancing, heterogeneous, interpolar, and exophytic, and with faint central T2 hyperintensity without clear evidence of vascular involvement or lymphadenopathy (Figures 2-3). Surgery and urology were consulted and an appendectomy with a total right nephrectomy was performed without complication the following morning. Biopsy revealed renal cell carcinoma (Figure 4). Shortly after surgery, the patient developed hypoxia requiring supplemental oxygen with tachypnea. On postoperative Day 1, he reported dyspnea with exertion; however, the team was unable to discern whether this was to be expected postop dyspnea with exertion versus dyspnea due to another cause. There was no significant calf or thigh swelling, erythema, edema, tenderness, or palpable cords. The patient did not report a cough. A pulmonary ventilation and perfusion scan was ordered due to concern that contrast would be nephrotoxic after a recent nephrectomy, which revealed findings suggestive of a pulmonary embolus. On postoperative Day 2, the patient was started on heparin at a prophylactic dose. The patient did not report chest or pleuritic pain, cough, hemoptysis, or jugular venous distention. Chest X-ray revealed minimal left basilar atelectasis. Laboratory analysis revealed decreased platelet count and decreased renal function as expected after right total nephrectomy. The patient continued to require supplemental oxygen after her operation. Confirmatory chest CT pulmonary angiogram revealed large saddle emboli with smaller thrombi extending to the segmental and subsegmental branches of the right upper lobe, right middle lobe, lower lobe, and left upper lobes on postoperative Day 5. A heparin drip subsequently started (Figures 5-6). A thrombectomy was performed the following day; no additional complications were noted. 

## Discussion

What makes this case pertinent is how insidious a silent PE can be. This case report highlights the importance of keeping a PE high in the differential diagnosis even if there are no classic signs and symptoms of this condition. In medical literature, silent PEs can be as common as 43.1%, as found in one study from 2000; another study from 2010 found the incidence to be 32% [2,3]. In contrast to the clinical case presented above, those studies consistently demonstrated remarkable venous Doppler ultrasounds with signs of thrombi. The 2022 study was a single-center retrospective cohort study with selection criteria for patients with positive Doppler studies who were then eligible to receive a CT pulmonary angiography and CT venography to look for evidence of a silent PE [2]. The 2010 study was a systematic review of 28 studies, the selection criteria included if the PE was stated to be “asymptomatic” [3]. While the 2022 study was able to correlate PE with more proximal deep vein thrombosis (i.e. iliofemoral), the 2010 study cited the advantages of routine PE screenings due to the high prevalence of silent PEs 33.3% [2,3].

It has previously been written that pulmonary emboli are associated with an acute decrease in platelets as well as tachycardia [4-9]. However, an acute increase in oxygen requirement has not been an additional consideration noted in our literature search. In addition, the modified Wells and the modified Geneva score do not take into consideration platelet counts or increased oxygen requirement necessitating supplemental oxygen [5,9]. The patient did not develop tachycardia at the exact time when they required additional oxygen supplementation. The patient presented above had a modified Wells score and modified Geneva score that were both elevated due to cancer and recent surgery. This suggests that pulmonary emboli should remain high in the differential diagnosis for relatively asymptomatic patients with increased oxygen requirements [5,9].

## Conclusions

Despite never having prior hemodynamic compromise, classic PE symptoms, or positive venous Doppler readings, the subject of this report was found to have a massive saddle PE. Fortunately, the patient was able to receive timely treatment, but this report stresses how insidious a silent PE can be. One needs to be vigilant in regard to patients that have high-risk factors (malignancy, recent surgery, acute illness) with signs of acutely increased oxygen requirement and thrombocytopenia.
